# Annotations

**Published:** 1888-07-21

**Authors:** 


### Annotations.

**The Dorset County Hospital.**

It was hoped some time ago that the wranglings and contentions which have so long given the Dorchester County Hospital an unenviable notoriety, were coming to an end. Not so, however. The local newspapers still continue to run over in all direction with the floods of verbiage which are poured out on both sides. Whether this kind of thing is really worthy of the opposing parties is another question. Certain classes of persons undoubtedly take delight in a war of words, but we doubt if they belong to the stronger-minded order of creation. Now, there must be a right and a wrong to all this squabbling, although it does not follow that either side has the whole right or the whole wrong. It may not, indeed, be possible now to decide whether of the two parties has the more right on its side. But there is such a thing as burying the hatchet; and it seems to us, from a more extended and personal knowledge of the case, that that course should now be adopted. If there were any charge against Dr. Lush and his colleague of such a nature that its proof would render either or both of them unfit for the post of physician or surgeon, then undoubtedly compromise would be impossible, and the only course open would be one of fighting to the bitter end. But nobody suggests that this is the *status quo*. The truth is, that a quarrel has been got up; the opposing parties are equally matched in everything but numbers, and neither party will give in. The numbers have called upon Dr. Lush to resign; the tough and dialectically skilful minority have called upon the numbers to resign. Is there any need for either party to submit to such humiliation? Surely something less than blood may satisfy both. We are aware that both parties are English, and your average Englishmen hates to be beaten. That is a feeling we entirely sympathise with; but if we were to see twenty hounds at a fox, and the fox should contrive by any me ıns whatever to keep them at bay for twenty minntes, we would whip off the hounds and give plucky Reynard another chance for his life. The minority cannot give in because they are a minority, but the majority, because they are a majority, can very properly do so with all the grace in the world. Quarrelling about matters of real moment may be not only justifiable, but respectable, though even then it should come to an end within the limits of decency. But to keep up a public wrangle before the eyes of the whole county for months about little or nothing, is only worthy of children, not of men.

**The Massacre of the Innocents.**

The fourth annual report of the Society for Preventing Cruelty to Children proves very painful reading, though the committee thankfully declare that their noble work is becoming more and more successful. The fact that they have procured convictions in 96 per cent. of the cases they have brought into court is in itself satisfactory, not so much because a certain number of brutal men and women—parents and others in authority—have been punished for cruelties and offences that cannot be named, against helpless and innocent children, as because the report of these punishments serves to deter others from similar crimes. Prosecution is a good and necessary thing, but the committee are even more pleased when they can check cruelty without resorting to the law courts. And they are often able to do so; the receipt of their formula of warning that if cruelty is persisted in, action will be taken, has often been enough to save a child from further torture. Cruelty and cowardice go together: the father who will break his child's bones, the mother who will rasp the flesh off her baby's back, have the most intense fear of imprisonment and of that wholesome tonic, hard labour. There are two things against which the society protests most strongly. One is the disabilities under which wives at present labour, as compared with unmarried mothers. An illegitimate child has, as things at present stand, more chance of protection from paternal brutality than one born in wedlock. On the other hand, the poor little love-child—often most wrongly so entitled—is frequently subjected to great cruelty because of the circumstances of its birth, from parents in whose degraded minds some strange distorted views of virtue linger. The other special danger to child-life lies in the practice of infant insurance. When a child's death will bring in a few pounds to a greedy and selfish parent, it has but small chance of prolonged life, and the only care that will be taken of it will be that it shall die in such a fashion that a doctor's certificate will not be refused, for this would invalidate the claim to the insurance money. In such cases starvation, and with infants, improper feeding, is resorted to, though deeds of active cruelty are not wanting. It will be said that poverty and the impossibility of providing for the wants of a large family offer some excuse for at least neglect. Alas! the worst cases have been found where real want was unknown, though a drunken and self-indulgent father spent large wages on his own vile pleasures, and where the family consisted of only one or two children. But we do not wonder at the last fact. Systematic slow murder will always keep down the number of a man's children.

**An Apostle of Long Life.**

Dr. B. W. Richardson may properly be designated the "apostle of long life." Every medical man should, of course, be, and is in a more or less complete sense, a member of this apostolate. Some people doubt whether long life be really an advantage. Undoubtedly it is advantageous, because in order to live long a man must be fairly healthy, and whether mere duration of existence be desirable or not, no one questions the desirability of health. As a matter of fact, however, the ordinary man or woman is still human enough to wish to live as long as possible. The mere philosopher is in such a decided minority on this question that for practical purposes he may be left out of account. Can Dr. Richardson really tell the world anything of actual moment in the way of making life longer? Undoubtedly! He both can and does. Last week, at the Sanitary Institute, he propounded principles which, if thoroughly and generally observed, would add considerably to the average duration of life. Unfortunately these principles, though true, are not new, and being old, it is to be presumed that they are unpopular, otherwise they would long ago have come into general use. Moreover they possess the disadvantage of requiring the use of both brains and self-denial by those who would put them in force. Now, the average mortal, though possessed of brains and of the power of self-denial, has a great objection to the use of both. If Dr. Richardson would invent a "long-life pill" he would beat Beecham or Holloway out of the field. But when he tells the world of such common-place expedients as the virtue of continency, maintenance of balance of bodily functions, perfect temperance, purity from implanted or acquired disease, and other mere common-sense and obvious deductions from experience, the world laughs him to scorn. It is with the body as with the mind and the soul. Everybody is for short and easy methods. A pill for the body, a formula and a creed for the soul: these are the highways to popularity and success. Nevertheless, this of Dr. Richardson's is the only way of life for the material organism: Sense, self-denial, well-doing. Is it not also the only way of a higher life? And does not this very unity and uniformity suggest that these must be the everlastingly and philosophically true ways. The perfect way of life is in itself simple and natural, and may be understood and followed by a child. The wilfulness and selfishness of human beings gives to life all its complexity and all its misery. It is a pity that Dr. Richardson and other men of simple and philosophic mind constitute so insignificant a minority.

